# Major Role of the IL17/23 Axis in Psoriasis Supports the Development of New Targeted Therapies

**DOI:** 10.3389/fimmu.2021.621956

**Published:** 2021-02-25

**Authors:** Hélène Bugaut, Sélim Aractingi

**Affiliations:** ^1^ Faculté de médecine, Université de Paris, Paris, France; ^2^ Service de dermatologie, Hôpital Cochin, Paris, France; ^3^ U932 Immunité et cancer, Institut Curie, Paris, France; ^4^ Equipe Biologie cutanée, Institut Cochin, Inserm, UMRS1016, Paris, France

**Keywords:** IL23, IL17, psoriasis, skin, Th17

## Abstract

Psoriasis is a frequent, chronic disease characterized by cutaneous inflammatory plaques and/or arthritis. It may be associated with few other diseases, mainly Crohn’s disease and metabolic syndrome. The medical and psychosocial burden of psoriasis remains high even since biological treatments arose, stressing that efforts to decipher its physiopathology are constantly needed. Tumor-necrosis factor α, interleukin (IL) 12 and IL17 have been previously associated with psoriasis and successfully targeted by monoclonal antibodies. IL17 in particular has been initially described as a T helper (Th) 17—produced cytokine, but it is now established that other cell types, such as γδ T lymphocytes, Mucosal-Associated Invariant T (MAIT) cells and Innate Lymphoïd Cells (ILC) 3 are also important sources of IL17 in the skin in response to inflammatory stimuli. Th17 phenotype has been shown to be stabilized by IL23, which is synthetized by macrophages and dendritic cells in response to Toll Like Receptors and C-type Lectin Receptors stimulation. Recent data also reported a crucial role for IL23 in MAIT17 and ILC3 homeostasis. Genome-wide association studies have found a significant link between IL23 receptor polymorphism and psoriasis susceptibility. IL23 signals through Janus kinase 2 and Tyrosine kinase 2, against which specific inhibitors are currently being tested. Monoclonal antibodies against IL17 and IL23 are only the beginning of a new avenue in psoriasis treatment. This review focuses on the molecular basis underlying IL23/IL17 axis blockade in psoriasis, and on future targets in this pathway.

## Introduction

Psoriasis is a chronic inflammatory disease involving the skin and/or the joints. Psoriasis prevalence in adults ranges from 0.51 to 11.43% worldwide (1) but is mainly considered to affect 2–3% of the population, with similar frequency in males and females (2). Skin lesions are featured by relapsing cutaneous erythro-squamous patches in its most frequent form, namely psoriasis vulgaris (PV). These will target electively peculiar locations such as scalp, palms and soles but also sacrum or large folds. Arthritis (PA, psoriatic arthritis) is associated to skin lesions in 1 to 15% of psoriasis patients (3). Of note, PA may either concern the peripheral articulations being therefore close to the characteristics of rheumatoid arthritis or in the contrary involve the spine, close to ankylosing spondylitis.

Psoriasis belongs to the spectrum of autoinflammatory diseases. Even in psoriasis without PA, its medical and psychosocial burden is high. Indeed, several studies using robust tools indicate that in all countries psoriasis may severely affect the quality of life (QOL) of these patients (4). In addition, psoriasis is significantly associated with metabolic syndrome, cardiovascular comorbidities and more rarely Crohn’s disease (5).

Since the 1990s, genetic and immunological studies have impressively dissected the mechanisms of psoriasis. Briefly, the disease appears to result from the interaction of genetic background and environmental triggers. Susceptibility loci mainly belong to HLA class I and II genes but also to various genes implicated in interleukin (IL) 17, IL23 and nuclear factor-kappa B (NFκB) pathways. In susceptible individuals, especially in response to physical traumatisms, autoantigens from keratinocytes, such as DNA, conjugate with anti-microbial peptides like cathelicidin/LL-37 (6), and activate plasmacytoid dendritic cells (pDCs) in the dermis. pDCs then secrete type I interferon and tumor-necrosis alpha (TNFα), which will activate classical dendritic cells (cDCs). These cDCs will produce IL12 and IL23, and skew the education of naïve T cells into T helper (Th) 1, Th17 and Th22 cells. Tumor-necrosis alpha (TNFα), IL17, and IL22 produced by these CD4+ T cells will then promote secretion of pro-inflammatory chemokines by keratinocytes, proliferation of epithelial cells and hyperkeratosis, and the recruitement of more inflammatory immune cells, accounting for the erythemato-squamous clinical lesions (7).

Resulting from this knowledge, targeted therapies have been a turnover in the management of psoriasis. TNFα blocking agents have initially paved the way, followed by monoclonal antibodies directed against IL12/23, IL17, and IL23. This highlights the crucial role of the IL17/23 cytokine pathway in psoriasis pathogenesis. Constant efforts are required to decipher the molecular mechanisms behind this disease, since new treatments are still needed for refractory and severe cases.

## Genetic Variants Highlight Critical Immunological Pathways

Heritability might account for as much as 68% of psoriasis susceptibility in Europeans (8). The first genetic linkage analyses in familial psoriasis (9) demonstrated the role of major histocompatibility complex alleles, mainly HLA-C*06:02, and were further confirmed by genome-wide association studies (GWAS) (10). Further insights into psoriasis genetics confirmed the importance of several immunological pathways among variants (11). Various mutations activating the pro-inflammatory NFκB pathway downstream of IL17 receptor (IL17R), such as in *TRAF3 Interacting Protein 2* (*TRAF3IP2*), which encodes ACT1, a protein that allows signal transduction from the IL17R and downstream activation of NFκB, in *CARD14* (an activator of NFκB), and in TNFAIP3 (tumor necrosis factor alpha induced protein 3, also called A20) and TNFAIP3 Interacting Protein 1 (TNIP1), have also been associated with an increased risk of developing psoriasis by GWAS studies, in Asian and Caucasian populations (10, 12–20).

Th17 lymphocytes are a major source of IL17, and they require IL23 to maintain their phenotype and to produce large amounts of IL17 (21–24). Polymorphisms in both subunits of IL23, IL23A(p19) and IL12B(p40), and in IL23R, have been associated with an increased risk of psoriasis in North Americans, Europeans and Asians (10, 12, 25). Signaling downstream of IL23 requires the signal transducer and activator of transcription protein 3 (STAT3). Polymorphisms have also been found by a GWAS meta-analysis in this gene (13). *Interferon regulatory factor 4* (*IRF4*), another gene whose variants are associated with psoriasis (13), encodes a transcription factor that binds to the IL17 promoter and regulates Th17 pathogenic properties (26, 27). IRF4 also drives the differentiation of conventional dendritic cells (cDCs) into cDC2, which produce IL23 and promote Th17 (28, 29).

Eventually, clues for the implication of other sources of IL17 than Th17 in psoriasis pathogenesis may also be suggested by the association of *Runt-related transcription factor 3* (*RUNX3*) polymorphisms to psoriasis susceptibility (13). RUNX3 is indeed a critical transcription factor for innate lymphoid cells (ILC), in particular for ILC3, the IL17 producing subset (30).

The vast majority of available genetic association studies highlight the role of the immune system in the pathophysiology of psoriasis. Even if some other genes such as IL-36 receptor antagonist (*IL-36RN*) are implicated (31, 32), the IL-17/23 axis seems to play a cardinal role.

## IL17 as a Central Effector in Psoriasis

An extensive amount of evidence now places IL17 as a key player in psoriasis pathogenesis (33, 34). In addition to genetic association studies, the efficacy of monoclonal antibodies targeting IL17 is a strong argument for the implication of this cytokine (35, 36).

Six isoforms of IL17 exist, and IL17A and IL17F are deemed to be the most pathogenic in psoriasis (37). IL17 receptors are heterodimers of IL17RA and a ligand specific subunit (IL17RB–E). IL17 receptors are widely expressed on epithelial cells (38). Upon the recognition of its ligand, IL17R recruits ACT1, which binds to tumor necrosis factor receptor 6 (TRAF6) (20). Downstream signaling involves mitogen activated protein kinase (MAPK), NFκB and C/EBPβ/δ pathways (19, 39–41). IL17 drives secretion of inflammatory chemokines, cytokines and antimicrobial peptides by keratinocytes, such as chemokine (C-C motif) ligand 20 (CCL20), IL-8 and β-defensin2 (42–45). IL17 indeed seems to play a key role in skin local immunity, as inborn deficiencies of IL17 or IL17R are responsible for chronic mucocutaneous candidiasis in humans (46). Pro-inflammatory mediators then recruit more Th17 lymphocytes, for example through CCL20/CCR6 signaling (47), and neutrophils, and increase local inflammation, resulting in the erythematous lesions characteristic of psoriasis.

Th17 cells were the first described source of IL17 (21, 48) and as such have been implicated in the pathogenesis of psoriasis (49). Th17, along with Th1, are found in the dermis of psoriatic lesions, and produce IL17 and IL22 (50), which in turn drives inflammatory and antimicrobial molecules secretion by keratinocytes (51). In mice, Th17 differentiate from naïve T CD4+ lymphocytes upon IL6 and transforming growth factor β stimulation; this process is amplified by IL1β and TNFα. Th17 cell survival and expansion depends on IL23 (21). IL23R is induced in Th17 by IL6 signaling through Janus kinases (JAK) JAK1, JAK2 and tyrosine kinase 2 (TYK2), STAT3 and RAR-related orphan receptor gamma t (RORγt) (52–54). IL23 signals through JAK/STAT3, resulting in enhancement of the Th17 phenotype (22). In humans however, Th17 require IL23 and IL1β for their differentiation (51). Keratinocytes also produce cytokines such as IL1β which amplifies Th17 generation (55).

Of note, Th17 are not the only source of IL17 in psoriasis. Other innate subsets, such as unconventional T cells, produce this key cytokine and might represent new therapeutic targets (56).

### Innate Cells Are Key Sources of IL17

Gamma delta T lymphocytes (Tγδ) are abundant innate-like T lymphocytes in the dermis. They can be divided into T-box expressed in T cells+ (T-bet)+, IFNγ-producing Tγδ and RORγt+, IL17-producing Tγδ (57–59). The IL17-producing subset predominates in the dermis, expresses IL23R, depends on STAT3 signaling and is a major source of pathogenic IL17 in psoriasis (60–62).

Mucosal-associated invariant T cells (MAIT) are recently characterized innate-like T lymphocytes which recognize metabolites produced by bacteria and fungi. They are abundant in barrier tissues and especially in the skin. Although rare in mice, they represent 1–10% of T lymphocytes in human blood, skin and intestine (63–65). In mice, they are also divided into MAIT1 and MAIT17 subsets, expressing T-bet and RORγt and producing IFNγ and IL17, respectively (66). MAIT17 rely on IL23 for their homeostasis and activation (67, 68) and are enriched in psoriatic skin lesions (64).

Another important type of unconventional cells are innate lymphoid cells (ILC). They have a lymphoid morphology, do not rely on recombination-activating genes (RAG) for their development, and lack myeloid, dendritic and T/B markers. Type 1 ILC encompass NK cells and ILC1, express T-bet and secrete IFNγ; ILC2 are characterized by GATA3 expression and IL5 and IL13 secretion; while ILC3 express RORγt and require IL23 to produce IL17 (69). ILC3 - like Th17 cells, IL17-producing Tγδ cells and MAIT17 cells - are increased in blood and cutaneous lesions of psoriasis patients (70, 71).

Several reports of IL17 secretion by neutrophils through extracellular traps production in psoriasis have been published (72–74). Neutrophils seem to express IL23R and RORγt (75), but their contribution to IL17 production in psoriasis is still largely unknown.

Eventually, keratinocytes themselves are able to produce IL17C, enhancing inflammation in psoriasis in an autocrine way (37, 44, 76, 77).

### IL23 Is a Key Regulator of IL17 Production

In many IL17-producing cell types, IL23 plays a pivotal role in IL17 secretion (78). IL23 induces Th17 phenotype in humans (51) or maintains this phenotype in mice (21–24). IL23 is required for IL17 production by skin Tγδ (62), MAIT17 (67, 68), ILC3 (69) and maybe by neutrophils (75). The receptor for IL23 is a heterodimer of IL23R, which signals through JAK2, and of IL12Rβ1, which signals through TYK2. Both activate STAT3, resulting in RORγt expression and IL17 secretion (22, 79).

It seems that in the gut, contrary to the skin, IL17 might play a protective role on the epithelial barrier, and its secretion seems to be at least partially IL23-independent (80). This difference may account for the worsening of Crohn’s disease symptoms in psoriasis patients treated with antiIL17, which is not found so far during IL23 blockade (81).

IL23 is mostly produced by cDC2 in mice, which correspond to CD1c+ DC in humans. cDC2 are driven by the transcription factor IRF4 and promote Th17 differentiation in mice and humans (29). This IL23 production depends on Toll Like Receptors and C-type Lectin Receptors stimulation, and neurogenic locus notch homolog protein 2 (NOTCH2) signaling, in different models of inflammation including psoriasis (51, 82, 83).

The whole IL23/JAK/STAT3/RORγt/IL17 pathway plays a central role in psoriasis pathogenesis and is a key target of many recent and developing treatments for psoriasis.

### Targeting the IL23/IL17 Axis in Psoriasis

**Figure 1:**
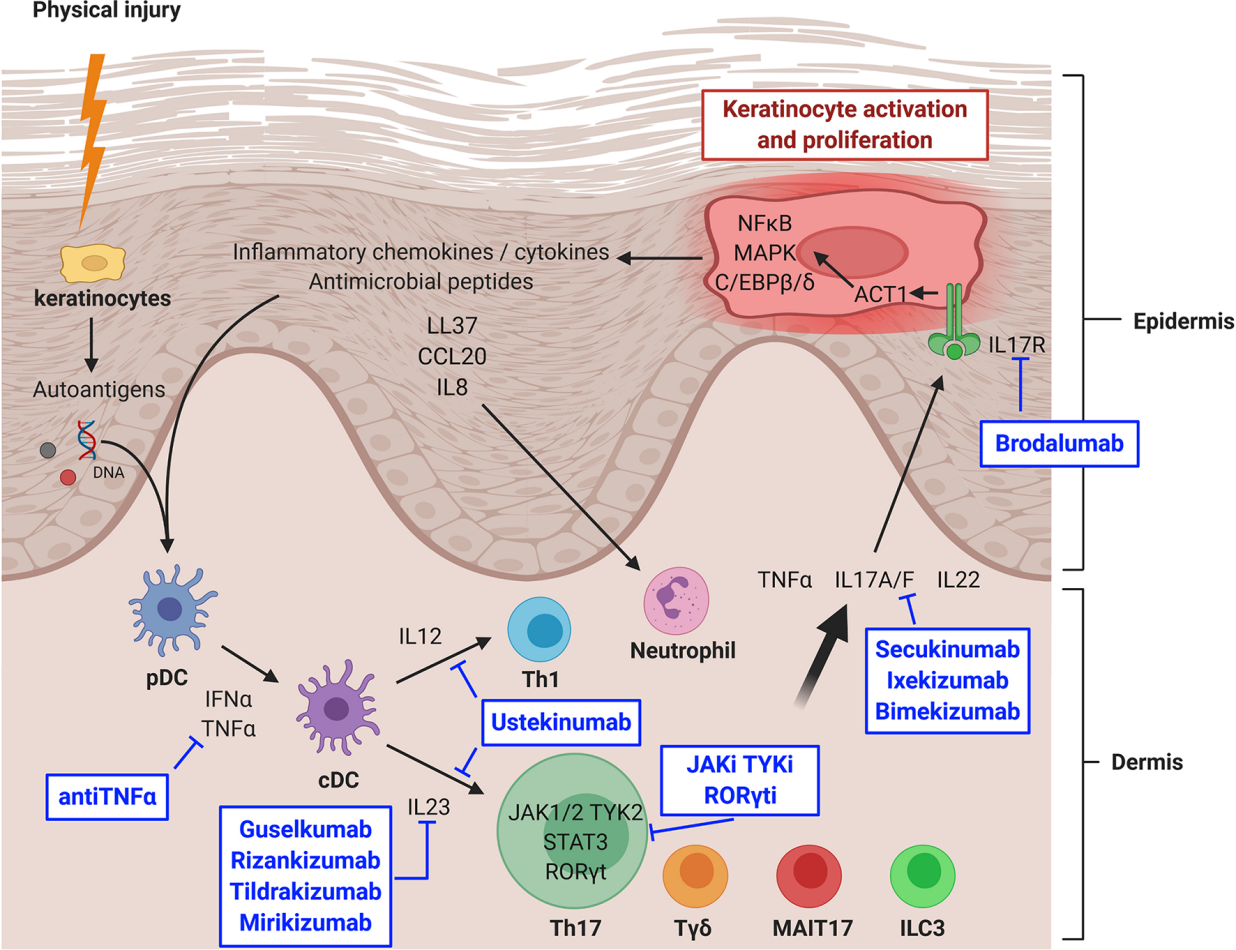
IL23/IL17 axis in psoriasis and targeted therapies. CCL20, chemokine (C-C motif) ligand 20; cDC, classical dendritic cell; IFNα, interferon alpha; IL, interleukin; IL17R, IL17 receptor; ILC, innate lymphoid cell; JAK, Janus kinase; JAKi, JAK inhibitor; MAIT, mucosal associated invariant T cell; MAPK, mitogen activated protein kinase; NFκB, nuclear factor-kappa B; pDC, plasmacytoid dendritic cell; RORγt, RAR-related orphan receptor gamma t; RORγti, RORγt inhibitor; STAT, signal transducer and activator of transcription protein; Tγδ, gamma delta T lymphocyte; Th, T helper lymphocyte; TNFα, tumor necrosis factor alpha; TYK, tyrosine kinase; TYKi, TYK inhibitor. Created with BioRender.com.

The development of new psoriasis treatments has nicely demonstrated *in vivo* the essential role of the IL23/IL17 axis in psoriasis (**Figure 1**). Ustekinumab, an antip40 (common to IL12 and IL23) antibody, represented the second generation of monoclonal antibodies developed in psoriasis after antiTNFα antibodies. It induces a nonspecific inhibition of Th1 and Th17 with a high efficiency (Psoriasis Area Severity Index improvement ≥ 75% (PASI75) at week 12: 67%), but that is reached slowly, usually in 3–6 months (84–86).

More recently, a third generation of monoclonal antibodies became available: secukinumab and ixekizumab targeted IL17A, whereas bimekizumab blocked both IL17A and IL17F and brodalumab inhibited IL17R. Their efficacy was also high (PASI75 at week 12: 77–86%) but reached much faster, in 1–3 months (35, 36, 87–89). However, unexpected flare-ups of Crohn’s disease happened in a minority of patients, whereas it was not the case during TNFα and IL12/IL23 inhibition (81, 90). Even if this over-risk is not fully confirmed (91), several studies now suggest that IL17 might play a protective role in the gut, where secretion by Tγδ and ILC3 might predominate, whereas IL17 is endowed with pro-inflammatory functions in the skin (44, 80, 92). Other expected side effects include diffuse candidiasis, as suggested by studies from inborn errors in IL17 signaling (46). A warning about suicide risk restricted to brodalumab (93) has been described but does not seem to be confirmed by more recent follow-ups.

The latest generation of monoclonal antibodies in psoriasis is represented by specific antiIL23 treatments such as guselkumab, rizankizumab, tildrakizumab and mirikizumab. Their efficacy is very high (PASI90 at week 16: 67–75%) and reached as quickly as when using antiIL17 antibodies, but without the previous side effect of IBD flare (94–99).

New therapeutic strategies in psoriasis now tend toward small molecules targeting JAKs, in order to prevent signaling downstream of IL23 and IL6. Tofacitinib, which blocks JAK1, JAK2 and JAK3, is tested in several clinical trials (100–105). Results are interesting but side effects, especially cytopenias, are pushing toward more selective JAK inhibitors (106). Specific TYK2 inhibitors are also under development, with an encouraging phase II trial (107), and several phase III trials ongoing (ClinicalTrials.gov identifiers NCT04036435, NCT03924427, NCT04167462, NCT03624127, and NCT03611751). Masitinib, the TYK c-kit inhibitor, is also undergoing a phase II trial (ClinicalTrials.gov identifier NCT01045577).

Inhibitors of RORγt are also under development, with an ongoing phase II trial (ClinicalTrials.gov identifier NCT04207801) while another one was terminated for adverse events (ClinicalTrials.gov identifier NCT03329885). RORγt inhibition could be relevant as it does not seem to affect Tγδ nor ILC3, which could spare the protective role of IL17 on the intestinal barrier (78, 108). Concerns about a risk of deep immunosuppression have been raised since RORγt is required at the early stage (double positive stage) of thymic development for all T lymphocytes (109, 110). Opportunist candidiasis and mycobacterial infections might also be a concern, since they are encountered in patients with inborn deficiencies in RORγt (111). Finally, conditional knock-out mice for RORγt develop lymphomas (112), which are thus closely monitored in clinical trials.

A promising strategy might be to use the topical route to avoid these potential serious side effects. Topical tofacitinib has shown promising results in a phase II trial (113). A topical formulation of a JAK1 and TYK2 inhibitor is currently undergoing a phase II trial (ClinicalTrials.gov identifier NCT03850483). Topical RORγt inhibitors are still in phase I or preclinical development (114, 115).

## Conclusion

IL23/IL17 axis plays a crucial role in psoriasis. Innate-like sources of IL17, such as Tγδ, MAIT and ILC3 are broadening the scope of pathogenic cells beyond classical Th17. Therapeutic targets now encompass IL23, JAK, RORγt and IL17 steps in this pathway, opening new avenues for resistant psoriasis treatment.

## Author Contributions

HB had written the first draft of the manuscript. SA had reviewed the manuscript extensively. All authors contributed to the article and approved the submitted version.

## Conflict of Interest

The authors declare that the research was conducted in the absence of any commercial or financial relationships that could be construed as a potential conflict of interest.
